# Elevated ZNF703 Protein Expression Is an Independent Unfavorable Prognostic Factor for Survival of the Patients with Head and Neck Squamous Cell Carcinoma

**DOI:** 10.1155/2015/640263

**Published:** 2015-04-29

**Authors:** Hang Yang, Wen-Qi Jiang, Ye Cao, Yong-An Sun, Jing Wei, Xin An, Ying-Chun Zhang, Ming Song, Shu-Sen Wang, Zhong-Yu Yuan, Rou-Jun Peng, Tan-Huan Chen, Li-Ren Li, Yan-Xia Shi

**Affiliations:** ^1^Sun Yat-Sen University Cancer Center, State Key Laboratory of Oncology in South China, Collaborative Innovation Center for Cancer Medicine, Guangzhou 510060, China; ^2^Department of Medical Oncology, Sun Yat-Sen University Cancer Center, Guangzhou 510060, China; ^3^Clinical Trial Center, Sun Yat-Sen University Cancer Center, Guangzhou 510060, China; ^4^Department of Neurology, Peking University First Hospital, Beijing 100034, China; ^5^Department of Medical Oncology, Guangxi Traditional Chinese Medical University, Ruikang Hospital, Nanning 530011, China; ^6^Department of Pathology, Sun Yat-Sen University Cancer Center, Guangzhou 510060, China; ^7^Department of Head and Neck Surgery, Sun Yat-Sen University Cancer Center, Guangzhou 510060, China; ^8^Department of Oncology, Huizhou Municipal Central Hospital, Huizhou 516001, China; ^9^Department of Colorectal Surgery, Sun Yat-Sen University Cancer Center, Guangzhou 510060, China

## Abstract

*Aim*. Data from The Cancer Genome Atlas (TCGA) show that the ZNF703 gene amplifies and overexpresses in head and neck squamous cell carcinomas (HNSCC). However, the clinical relevance of this observation in HNSCC is unclear. The purpose of this study was to clarify the expression of ZNF703 protein and its prognostic effect on HNSCC. *Methods*. Two hundred ten HNSCC patients from Sun Yat-Sen University Cancer Center with complete survival follow-up were included in this study. Tumor samples from primary sites were collected. The expression of the ZNF703 protein was tested by immunohistochemistry (IHC). *Results*. The high expression of ZNF703 in HNSCC tumor tissues was significantly higher than that of the matched noncancerous tissues (48.6% versus 11.6%, *P* < 0.001). ZNF703 overexpression was correlated with tumor position (laryngeal carcinoma) and recurrence (all *P* < 0.05). Multivariate analysis revealed that ZNF703 protein overexpression was an independent prognostic factor (*P* = 0.022, hazard ratio = 1.635, 95% CI 1.073–2.493) in HNSCC patients. *Conclusion*. ZNF703 overexpression is associated with adverse prognosis in HNSCC, which might be a novel biomarker of HNSCC.

## 1. Introduction

Head and Neck Squamous Cell Carcinoma (HNSCC) is the sixth most common malignancy in the world, with approximately 600,000 newly diagnosed cases per year [1, 2]. HNSCC is a group of fairly heterogeneous tumors that can occur from the base of the cranium to the clavicles. On the basis of anatomic sites, HNSCC can be divided into various types; the paranasal sinuses, nasal cavities, nasopharynx, orbits, oral cavity, oropharynx, hypopharynx, and larynx make up 90% of HNSCC and the 5-year survival rate of all HNSCC is approximately 50% [3]. Patients diagnosed at an early stage can have a superior quality of life and life expectancy with surgery alone [3]. The majority of patients are diagnosed at an advanced stage, whose prognosis is extremely poor [4]. For patients in stage IV, the long-term survival of HNSCC patients is only approximately 15 months [5]; the high recurrence rate highlights the need for a cure. Despite advances in diagnosis and treatment, the survival rates for many types of HNSCC have improved little over the past forty years [2, 3]. Therefore, a deeper understanding of HNSCC pathogenesis is needed to promote the development of improved therapeutic approaches.

In recent years, the development of high-throughput sequencing technology provides the best opportunity for exploring fundamental tumorigenic mechanisms. According to The Cancer Genome Atlas (TCGA) database, which included 4000 cases and more than 20 tumor types, ZNF703 is one of the most frequently altered genes in the pan-cancer group. In HNSCC, the incidence of an increased ZNF703 copy number was 28%, and the high-level of gene amplification accounted for 7.0% [http://www.cbioportal.org/public-portal/].

The zinc finger protein 703 (also known as ZPO1, ZEPPO1) gene is located on chromosome arm 8p12. The ZNF703 protein belongs to the NET (Noc/Nlz, Elbow, and Tlp-1) family, which plays an important role in the embryonic development of zebrafish [6] and Drosophila [7]. It has been shown that ZNF703 gene amplification stimulates migration and proliferation while reducing cell to cell adhesion [8–10] and is speculated to be associated with poorer outcomes [9–12] in breast cancer. Two studies from China also discovered that ZNF703 acts as an oncogene that accelerates malignant progression in gastric cancer [13] and is associated with worse prognosis in colorectal cancer patients [14]. As a consequence, we propose that ZNF703 may also play an important role in the pathogenesis of HNSCC. However, we are not aware of any reports about ZNF703 in HNSCC until now. Thus, we conducted this retrospective study to explore the expression of ZNF703 protein and its clinical relevance in HNSCC.

## 2. Materials and Methods

### 2.1. Patients and Tissue Specimens

A total of 210 HNSCC patients (160 male and 50 female) between January 2001 and December 2008 with complete survival follow-up documents and available tumor samples were enrolled in our study. All patients had undergone surgery at Sun Yat-Sen University Cancer Center, Guangzhou. The exclusion criteria were as follows: (i) accepted neoadjuvant radiotherapy and/or chemotherapy before surgery, (ii) having distant metastasis at the time of diagnosis (stage IVc), (iii) incomplete records, (iv) having other malignant tumors at any time during the treatment and follow-up, (v) with severe complications before treatment. The TNM stage was reclassified on the basis of the American Joint Committee TNM Staging system (7th ed., 2010). All patients were followed up until April 2014. Two hundred ten paraffin-embedded specimen blocks and 43 matched adjacent noncancerous tissue blocks (as control group) were obtained from the pathology department of Sun Yat-Sen University Cancer Center. All the slides were reviewed to reconfirm the diagnosis by two independent pathologists. All works were conducted in accordance with the Declaration of Helsinki (1964). Written and informed consent was obtained from every patient, and the study was approved by the ethics review board of Sun Yat-Sen University Cancer Center. Local or distant metastasis was confirmed by biopsy or radiological examination.

### 2.2. Immunohistochemistry (IHC) and Assessment

The paraffin sections (4 mm thick) were dewaxed and rehydrated, and endogenous peroxidase was first blocked with 0.3% H_2_O_2_ methanol. For antigen retrieval, the tissue slides were blocked in the 0.01 M citrate buffer (pH 6.0) in a microwave oven for 30 min. Nonspecific binding was inhibited with normal goat serum for 30 min. The primary ZNF703 antibody (ab155210, 1 : 400, Abcam, Cambridge, MA, USA) was used, and slides were put in a moist chamber overnight at 4°C. Subsequently, the slides were incubated with biotinylated secondary antibody for 30 min at 37°C. Horseradish peroxidase was subsequently applied. Finally, Meyer's hematoxylin was used for nucleus counterstaining. The negative control was obtained by omitting the primary antibody.

All immunostaining slides were judged by two independent pathologists without knowledge of the clinic-pathologic information. The specific intensities of staining were identified as follows: 0 = none; 1 = weak; 2 = moderate; 3 = strong. The proportion scores were as follows: 0 = none; 1 = 1–10%; 2 = 11–50%; 3 = 51–80%; 4 = >81%. The ZNF703 immunoreactive score for each tissue was calculated by multiplying the score of the staining intensity by that of the percentage of positive cells. If the discrepancy between the two pathologists' immunoreactive score was larger than 6, the tissue section was reevaluated by the two pathologists face to face.

### 2.3. Cut-Off Score Selection

**Figure 1:**
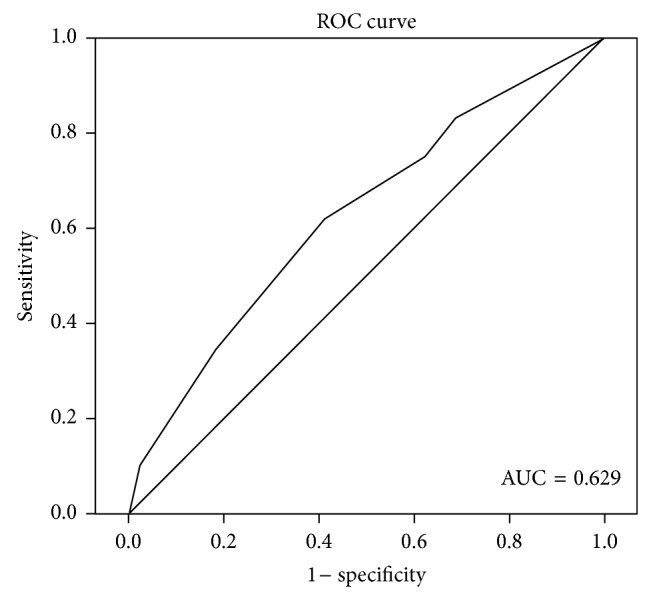
Selection of the cut-off score for positive expression of ZNF703 by ROC analysis. The area under the curve was 0.629, the point 3.5 could maximize the Youden Index [1.178, YI = sensitivity (0.544) + specificity (0.633)].

The median computed value of ZNF703 protein staining was 3, with a range of 0–12. The receiver operating characteristic (ROC) curve for the ZNF703 scope were plotted to select the potential cut-off score (Figure 1). The area under the curve was 0.629 (*P* = 0.001, 95% confidence interval = 0.553–0.705). As the optimal cut-off score, 3.5 point on the ROC curve could maximize the Youden Index [1.178, YI = sensitivity (0.544) + specificity (0.633)]. Thus, cases were divided into “low expression” (Allred score ≤ 3) and “high expression” (Allred score > 3) [15].

### 2.4. Statistical Analysis

The SPSS 19.0 software was used for statistical analyses. A receiver operating characteristic (ROC) curve and the median was used to determine the cut-off value to distinguish high or low ZNF703 expression. The relationship between clinic-pathological characteristics and ZNF703 expression was assessed by a *χ* ^2^-test. Overall survival time was defined as the duration between the time of disease diagnosis and the time of death. The Kaplan-Meier method was used for survival analysis. The Log-Rank test was conducted for differential survival analysis. Additionally, the Cox test was performed for multivariate analysis. The statistical tests were two-sided probability tests. Statistical significance was defined as less than 0.05.

## 3. Results

### 3.1. Clinical Features of Patients

The clinical features of the 210 patients are listed in Table 1. The TNM stage of the patients ranged from stage I to IVb. The median age was 53.5 years (range 25–86 years). All the patients had undergone complete excision with or without unilateral/bilateral neck dissection and accepted adjuvant radiotherapy according to the NCCN guideline. During the follow-up period, there were 132 (62.9%) local and distant recurrence events and 90 patients (42.9%) died. The median overall survival time (OS) was 80.4 (2.6–169.6) months, The 1-, 3-, and 5-year survival rates were 91.4%, 70.4%, and 64.1%, respectively. The disease-free survival (DFS) was in a range of 0.7–139.8 months, and the 1-, 3-, and 5-year disease-free survival rates were 63.8%, 46.7%, and 41.8%, respectively. Among the patients with recurrence, eighty-nine (67.4%) patients died by the end of follow-up, and the 1, 3, and 5-year survival rates were 86.5%, 52.7%, and 43.4%, respectively.

### 3.2. The Relationship between ZNF703 Expression and Clinicopathological Factors

**Figure 2:**
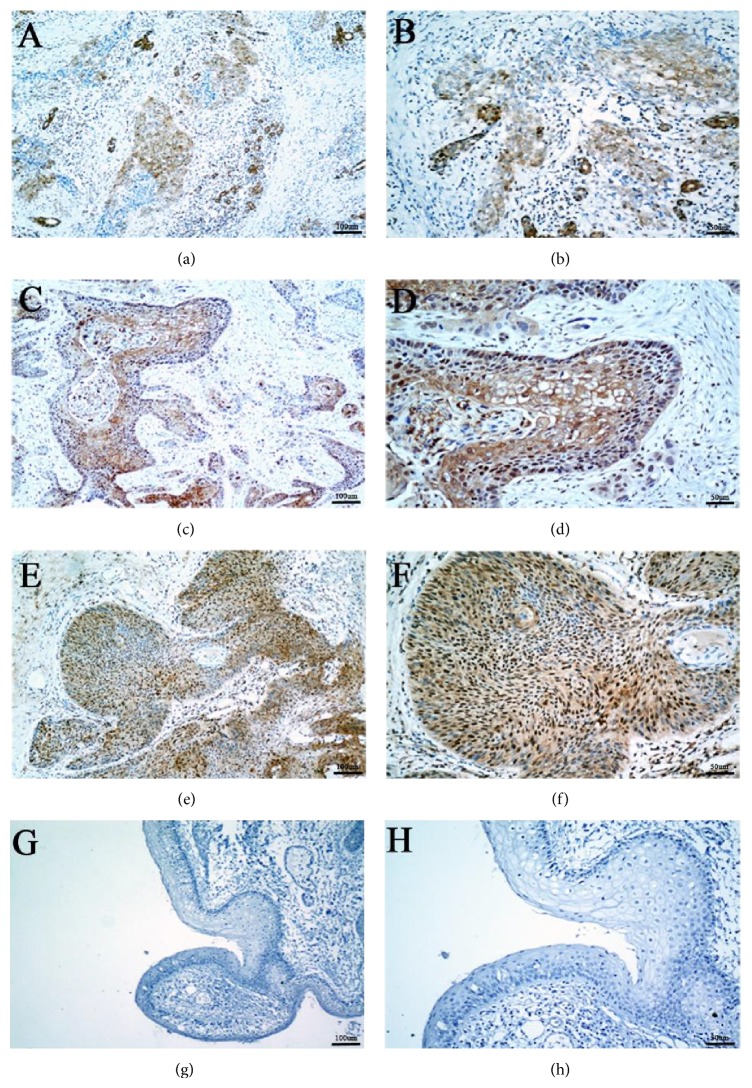
Expression of ZNF703 in tumor species and noncancerous species. ((a), (b)) Weak positivity in tumor cells, mainly stained in cytoplasm (100x and 200x, resp.). ((c), (d)) Moderate positivity in tumor cells, which are located in nucleus and cytoplasm (100x and 200x, resp.). ((e), (f)) Strong positivity in squamous carcinoma cells, mainly shown as nuclear staining. (100x and 200x, resp.). ((g), (h)) No ZNF703 protein staining was present in the noncarcinoma epithelium.

**Figure 3:**
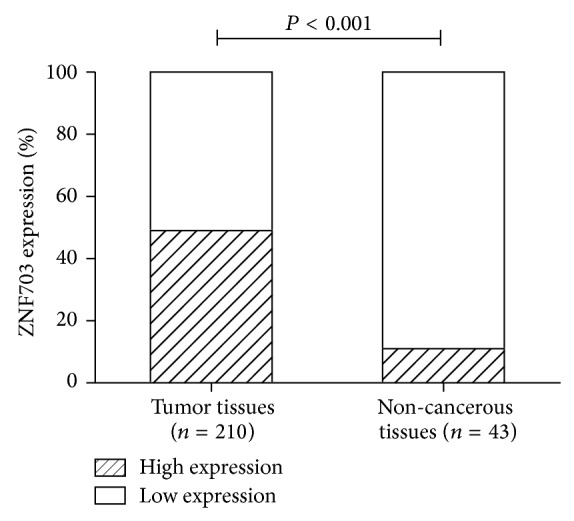
The expression of ZNF703 protein is elevated in tumor tissues and noncancerous specimens. The comparison of ZNF703 protein expression evaluated by IHC revealed that the percentage of high-expression cases in tumor tissues was significantly higher than that of the adjacent noncancerous matched tissue (*n* = 43).

The ZNF703 protein was located in the cytoplasm and nucleus, but mainly in the nucleus (Figure 2). The overexpression rate of ZNF703 protein was 48.6% in HNSCC tumor cells, it was significantly higher than that of the adjacent noncancerous squamous epithelium cells (Figure 3, 48.6% versus 11.6%, *P* < 0.001). The overexpression of ZNF703 was related to recurrence and primary tumor site but not related to T stage, N stage, age, gender, BMI, differentiation, and complications (Table 1).

### 3.3. The Relationship between ZNF703 Expression and Prognosis

**Figure 4:**
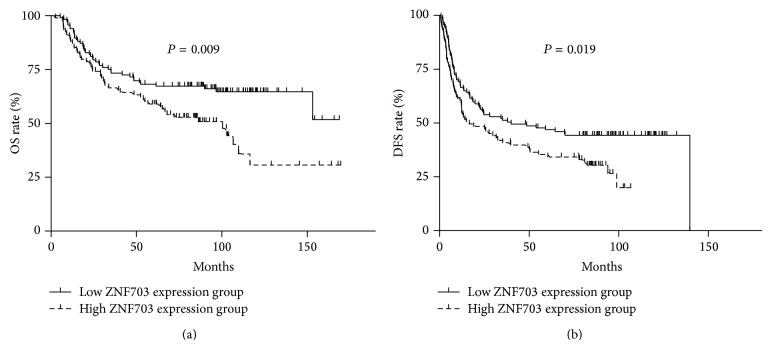
ZNF703 overexpression is associated with worse OS and DFS by Kaplan-Meier analysis.

Comparing to the ZNF703 low expression group, the ZNF703 high expression group had a significantly higher recurrence rate (71.0% versus 56.4%, *P* = 0.03) and mortality (52.7% versus 35.0%, *P* = 0.01). The mean OS and DFS in the ZNF703 overexpression group were 89.3 months (95% CI 74.3–104.3) and 43.7 months (95% CI 34.8–52.6), respectively. The 5-year OS and DFS rates were 58.9% and 34.2%, respectively, whereas in the ZNF703 low level group, the mean OS and DFS times were 117.7 months (95% CI 105.1–130.4) and 71.1 months (95% CI 59.7–82.5), respectively, and the 5-year OS and DFS data were 68.2 and 46.9%, respectively. In univariate survival analysis, a high ZNF703 protein level was an important prognostic factor for shorter overall survival (*P* = 0.009) and DFS (*P* = 0.019) (Table 2, Figure 4). It was also observed that male patients, patients who consumed tobacco and alcohol, and patients with advanced stage disease, complications, or poorer differentiation had an apparently shorter OS. Meanwhile, poorer differentiation, advanced stage disease, without neck dissection or adjuvant radiotherapy and laryngeal cancer were all associated with shorter DFS (Table 2). In the multivariate Cox proportional hazards model analysis, only ZNF703 overexpression (*P* = 0.022, hazard ratio = 1.635, 95% CI 1.073–2.493), advanced stage, complications, and alcohol consumption were independent prognostic factors in HNSCC patients (Table 3).

## 4. Discussion

A variety of genetic changes have been described previously in HNSCC, including frequent chromosome 8p amplification [16–21]. It has also been identified that amplifications at 8p12 occurred frequently in multiple cancer types. This was suggestive of the presence of an important oncogene in this region, and numerous studies have been carried out to identify the driver gene on 8p12.

There are five genes in this area:* ERLIN2, PR OSC, BRF2, RAB11FIP1, and ZNF703* [22–24]. Recent research showed that ZNF703 is amplified in approximately 15% of breast tumors [23, 25–27], which is only less than HER2 and cyclin D1 (CCND1) [26, 28, 29], especially in Luminal B molecular subtypes [9–11, 29]; thus, more attention has been focused on ZNF703.

In the TCGA database of HNSCC, the amplification rate of the ZNF703 gene is 7% [http://www.cbioportal.org/public-portal]. Our results also showed that the expression level of ZNF703 protein was significantly higher in HNSCC than in the adjacent noncancerous squamous epithelial cells, the overexpression rates were 48.6% and 11.6%, respectively (*P* < 0.001). Furthermore, ZNF703 overexpression was observed more frequently in larynx cancer and was correlated with higher risk of recurrence. The 5-year OS rate was 58.9% and 68.2% in the high and low expression groups, respectively. All these results indicated that ZNF703 may play a role in the tumorigenesis and metastasis of HNSCC and act as an oncogene.

Overexpression of ZNF703 was also found in other cancers, such as breast cancer, gastric cancer, and colorectal cancer. Zhang et al. reported that the positive expression rate of ZNF703 in early stage breast cancer patient was 91.3% [12]. Yang et al. found that the protein was also upregulated in invasive gastric carcinoma tissues [13] and colorectal cancer samples [14]. Interestingly, further studies reported that patients with higher N stage and high risk of tumor relapse and death had a higher level of ZNF703 expression (*P* < 0.05). This finding prompted researchers to consider whether the ZNF703 protein is a candidate factor for promoting HNSCC growth, progress and metastasis.

Although there is still no direct report about the pathogenic mechanism of ZNF703 in HNSCC, reports in other cancer types may help to understand its mechanism. Earlier studies also showed that ZNF703 stimulated mammary epithelial cells toward immortalization rather than differentiation and enhanced the invasive potential of breast cancer cells [9, 10]. The possible mechanism is thought to be involved in the EMT. The ZNF703 could downregulate E-cadherin and promote metastasis-associated p120-catenin isoform 1 expression. Subsequent data demonstrated that ZNF703 overexpression increased lung metastases in a mouse breast cancer model [8, 30]. It may also play a role in the activation of stem cell-related gene expression, leading to an increase in cancer stem cells [10]. On the other hand, it was reported to be involved in the activation of the Akt/mTOR signaling pathway, downregulated ERalpha, and the reduction of the antitumor effect of tamoxifen in breast cancer in vitro [12]. Based on these results we suggest that ZNF703 may also play an important role in HNSCC formation and progression.

In our study, we only detected ZNF703 protein expression with IHC, so there is no final conclusion about the status of ZNF703 gene amplification or mRNA levels in HNSCC. However, according to the TCGA database of patient tumor samples and the CCLE database of cell lines and other literature [9], the copy number and gene expression are highly positively correlated, *r* = 0.72 [http://www.cbioportal.org/public-portal].

On the basis of these results, we speculate that ZNF703 could be a valuable independent factor in HNSCC for predicting prognosis as well as a promising biomarker to help design optimal individual treatments.

## 5. Conclusion

On the one hand, our findings suggested that the overexpression of ZNF703 is common in patients with HNSCC and is related to the aggressive clinical course and poor prognosis. Therefore, more aggressive therapy and closer follow-up should be adopted in patients with ZNF703 overexpression. On the other hand, our findings also provide new clues for further exploration of new treatments in HNSCC. To the best of our knowledge, this is the first report about the expression of ZNF703 in HNSCC. However, due to the retrospective research and small sample size, further prospective clinical study and genomic, cellular function and mechanistic studies are needed to prove our conclusions.

## Figures and Tables

**Table 1:** Correlations between ZNF703 expression and clinicopathological characteristics of the patients.

Parameter	ZNF703 expression		*P* value
	Low (117)	High (93)	
Age ( years)			0.420
≤50	52	31	
51–60	34	32	
61–70	24	22	
>70	7	8	
Gender			0.093
Male	84	76	
Female	33	17	
BMI (kg/m^2^)			0.298
<20	22	24	
20 to <25	70	46	
≥25	25	23	
Tobacco consumption			0.202
No	53	34	
Yes	64	59	
Alcohol consumption			0.208
No	93	67	
Yes	24	26	
Histological differentiation			0.324
Well	76	51	
Moderately	31	31	
Poorly	10	11	
Complications			0.649
No	96	74	
Yes	21	19	
T stage			0.549
T1	68	45	
T2	31	30	
T3	12	13	
T4	6	5	
N stage			0.071
N0	87	67	
N1	19	12	
N2	11	10	
N3	0	4	
Cancer stage			0.362
I/II	80	58	
III/IV	37	35	
Tumor position			**0.026**
Oral cavity	100	68	
*Buccal mucosa *	*4 *	*8 *	
*Floor of mouth *	*9 *	*3 *	
*Anterior tongue *	*70 *	*41 *	
*Alveolar ridge *	*15 *	*14 *	
*Hard palate *	*2 *	*2 *	
Larynx	17	25	
*Glottis *	*12 *	*18 *	
*Supraglottic *	*5 *	*7 *	
Disease recurrence			**0.030**
No	51	27	
Yes	66	66	

BMI = body mass index.

**Table 2:** Univariate survival analysis of different parameters in 210 patients with HNSCC.

Variables	*P* value	
	OS	DFS
Male	**0.048**	0.310
Complications	**0.047**	0.312
Tobacco consumption	**0.013**	0.128
Alcohol consumption	**0.001**	0.087
Age > 70 or <45	0.512/0.313	0.514/0.761
BMI < 20 or ≥25	0.519/0.427	0.975/0.518
Poorer differentiation	**0.005**	**0.001**
Without neck dissection	0.705	**0.002**
ZNF703 high expression	**0.009**	**0.019**
Stage III/IV	**0.000**	**0.001**
Larynx cancer	0.509	**0.000**
Without adjuvant radiotherapy	0.308	**0.037**

**Table 3:** Multivariate analysis of different parameters in 210 patients with HNSCC by Cox proportional hazard mode.

Variables	OS		
	RR	95% CI	*P*
Male	0.955	0.466–1.955	0.899
**Complications**	0.583	0.358–0.951	***0.031***
Tobacco consumption	0.755	0.421–1.352	0.344
**Alcohol consumption**	0.574	0.358–0.922	***0.022***
Poor differentiation	1.245	0.798–1.942	0.334
**ZNF703 high expression**	1.635	1.073–2.493	***0.022***
**Stage III/IV**	2.661	1.689–4.190	***0.000***

Variables	DFS		
	RR	95% CI	*P*

Poor differentiation	1.129	0.761–1.674	0.546
Without neck dissection	1.447	0.974–2.149	0.068
**Without adjuvantradiotherapy**	1.656	1.077–2.546	***0.021***
**ZNF703 high expression**	1.419	0.999–2.016	***0.050***
**Stage III/IV**	1.782	1.205–2.634	***0.004***
**Larynx cancer**	0.543	0.351–0.842	***0.006***

RR = relative risk; CI = confidence interval.
